# Indole-3-carboxyaldehyde does not reverse the intestinal effects of fiber-free diet in mice

**DOI:** 10.3389/fendo.2024.1362711

**Published:** 2024-03-22

**Authors:** Mark M. Smits, Serafina I. L. Dreyer, Jenna E. Hunt, Anna K. Drzazga, Ida M. Modvig, Jens J. Holst, Hannelouise Kissow

**Affiliations:** ^1^ Department of Biomedical Sciences, Faculty of Health and Medical Sciences, University of Copenhagen, Copenhagen, Denmark; ^2^ Institute of Molecular and Industrial Biotechnology, Faculty of Biotechnology and Food Sciences, Lodz University of Technology, Lodz, Poland; ^3^ Novo Nordisk Foundation Center for Basic Metabolic Research, Faculty of Health and Medical Sciences, University of Copenhagen, Copenhagen, Denmark

**Keywords:** fiber-free diet, intestinal health, GLP-1 secretion, indole, bacterial metabolites

## Abstract

**Objective:**

Fiber-free diet impairs intestinal and colonic health in mice, in parallel with a reduction in glucagon like peptide-1 (GLP-1) levels. Endogenous GLP-1 is important for intestinal growth and maintenance of the intestinal integrity. We aimed to investigate whether fiber-free diet reduces luminal content of metabolites which, upon supplementation, could increase GLP-1 secretion and restore the adverse effects of fiber-free diet.

**Methods:**

Untargeted metabolomics (LC-MS) was performed on colonic content of mice fed a fiber-free diet, identifying a metabolite of particular interest: indole-3-carboxyaldehyde (I3A). We exposed cultured GLUTag cells to I3A, and measured cumulative GLP-1 secretion. Isolated colon perfusions were performed in male C57BL/6JRj mice and Wistar rats. I3A was administered luminally or vascularly, and GLP-1 was measured in portal vein effluent. Finally, female C57BL/6JRJ mice were fed chow or fiber-free diet, with I3A or vehicle by oral gavage. After 10 days, plasma GLP-1 (ELISA) and intestinal permeability (FITC-dextran) were measured, animals were sacrificed and organs removed for histology.

**Results:**

Mice fed a fiber-free diet had significantly lower I3A in their colonic content compared to a control diet (7883 ± 3375 AU, *p*=0.04). GLP-1 secretion from GLUTag cells was unchanged after five minutes of exposure to I3A. However, GLP-1 levels increased after 120 minutes of exposure to 1 mM (60% increase, *p*=0.016) and 5 mM (89% increase, *p*=0.0025) I3A. In contrast, 48 h exposure to 1 mM decreased GLP-1 secretion (51% decrease, *p<*0.001) and viability. In isolated perfused mouse and rat colon, I3A applied into the luminal or vascular side did not affect GLP-1 secretion. Mice fed a fiber-free diet tended to weigh less compared to chow fed mice; and the small intestine and colon were significantly smaller. No differences were seen in crypt depth, villus length, mucosal area, and intestinal permeability. Supplementing I3A did not affect body weight, morphology or plasma GLP-1 levels.

**Conclusions:**

Fiber-free diet lowered colonic content of I3A in mice. I3A stimulates GLP-1 secretion *in vitro*, but not in animal studies. Moreover, it has no evident beneficial effect on intestinal health when administered *in vivo*.

## Introduction

Dietary fibers, or non-digestible carbohydrates, play an important role in maintaining intestinal health. Specific characteristics, like solubility, viscosity, and fermentability, determine their function in the gastrointestinal system, including how they act on intestinal transit time, stool formation and microbiota composition (1). As a whole, dietary fiber intake has been linked to a significant reduction in colon cancer occurrence (2), and it reduces cancer-related (and all-cause) mortality (3). Moreover, low fiber intake associated with constipation and its consequences (4). Despite the documented importance of dietary fiber for GI health, the exact etiology of this benefit is still not completely understood.

Mice fed a fiber-free diet displayed reduced small intestine (SI) and colonic weight, shorted SI crypt depths and increased intestinal permeability (5). Furthermore, reduced dietary fiber consumption has been linked to increased sensitivity to intestinal injury in mice (6). Increased permeability following a fiber-free diet has been linked to an increase in mucus-degrading bacteria in the colon (7). Previously, we have shown that fiber-free feeding reduced the amount of extractable GLP-1 in the colon (5). Studies involving GLP-1 receptor knock-out mice and GLP-1 receptor antagonists support the findings of GLP-1 being necessary for normal intestinal growth (8–10), maintenance of the intestinal barrier function (11), and resilience to intestinal injury (12–16). These outcomes highlight the need to explore possible mechanisms underlying the reduced endogenous GLP-1 following a fiber free diet. It is well known that the GLP-1 secreting enteroendocrine L-cell is stimulated by nutrients (17), however studies have revealed that bile acids and metabolites produced by the microbiota, such as short chain fatty acids (SCFAs), are also L-cell secretagogues (18).

Conceivably, the reduction of GLP-1 could be mediated by the absence of fiber-derived microbial metabolites, in turn causing reductions in intestinal weight and crypt depth. We therefore performed untargeted metabolomics on intestinal contents from mice fed normal chow or a fiber free diet. We identified that fiber-free diet induced a significant decrease in the colonic content of indole-3-carboxyaldehyde (I3A), a specific indole-subtype. Indole has been shown to increase GLP-1 secretion *in vitro* (19), but whether this is true for I3A is unknown. Furthermore, I3A has been shown to improve epithelial homeostasis (20). We therefore set out to assess whether supplementation with I3A would lead to increased gut-hormone secretion and improvement of the histologic changes induced by the fiber-free diet.

## Materials and methods

### Metabolomics

Using samples from a previous study where mice were given fiber-free diet or chow (5), we performed untargeted metabolomics on intestinal content of the proximal and distal small intestine and colon. Samples were combined with NaOH and shaken at 1400rpm at 22°C for 10min, followed by purification Oasis HLB 3 cc Vac SPE Cartridges (Waters), eluted in 1ml methanol and dryed overnight by speed vacuum. The dried material was then suspended in LC-MS grade water and transferred to autosampler vials. 10μl of each sample was combined in one vial to form a quality control QC-pooled sample and a blank containing LC-MS grade water was included. Separation was performed on a Phenomenex Luna Omega C18 column (100 mm length x 2.1 mm internal diameter, and 1.6μm particle size). Analysis was carried out in full scan, negative ion mode on a Bruker Impact II quadrupole time of flight (Q-TOF) mass spectrometer. Features were integrated using XCMS online [PMID: 22533540]. The features were normalized to sum intensity, log2 transformed and Pareto scaled. MetaboAnalyst 4.0 was then used to identify differentially regulated features. 33 out of 65 significantly different features were putatively identified by matching MS/MS spectra to either the Metlin [PMID: 29381867] or HMDB [PMID: 29140435] databases.

### Cell studies

GLUTag cells, kindly provided by Prof. Drucker (University of Toronto, Canada), were cultured at 37˚C and 5% CO_2_ in Dulbecco’s modified Eagle’s (DMEM) 1885 (glucose 5.6 mmol/L), supplemented with 10% (v/v) fetal bovine serum, 1% (v/v) penicillin (10,000 U/ml)/streptomycin (10,000 μg/ml) and 200 mM Glutamax. Medium was changed every 3 days, and cells were trypsinized and reseeded at a 1:5 dilution when ~80-85% confluent. For the current study, passage numbers 23 to 32 were used.

#### Secretion studies

For secretion studies, GLUTag cells were plated on 24-well culture plates at 250.000 cells per well. When 80% confluence was reached (between 24 and 48 hours), cells were washed and starved for 30 minutes with freshly prepared Krebs-Ringer-HEPES (KRH) buffer (138 mM NaCl, 4.5 mM KCl, 4.2 mM NaHCO_3_, 1.2 mM NaH_2_PO_4_, 2.5 mM CaCl_2_, 1.2 mM MgCl_2_, and 10 mM HEPES, pH = 7.4). Then, cells were incubated with KRH with 5.6 mM glucose (baseline secretion), or the same buffer with I3A (Sigma-Aldrich, St. Louis, MO; cat. no 129445; a stock solution of 100 mg/mL was made in DMSO).

IA3 concentrations and duration of stimulation were experiment-dependent. Positive control (10 mM glucose, 10 µM forskolin, and 10 µM 3-isobutyl-1-methylxanthin) were included in each experiment, but not shown here. Supernatants were obtained and centrifuged (1500 g, 4˚C, 5 min) to remove any debris. The resulting supernatants were snap frozen and stored at -20˚C until analysis. All cell experiments were performed on at least two separate occasions with at least three technical repeats per experiment.

As GLP-1 from GLUTag cells is only partly amidated (21), we measured GLP-1 using radioimmunoassay using the side-viewing 2135-antibody, which captures all molecules containing the central sequence of GLP-1 (including both amidated and glycine-extended variants).

#### Calcium imaging studies

Intracellular calcium mobilization was measured in real-time using the Fluo-4 AM dye (Thermo Fisher Scientific). Cells were seeded on a 96-well plate (30.000 cells/well) and cultured for 24 hours. Prior to the experiment, cells were loaded for one hour in 0.2% Fluo-4 (dissolved in 20 mM HEPES buffered HBSS with 5.6 mM glucose, 1 mM CaCl_2_, 1 mM MgCl_2_ and 0.5% probenecid [Thermo Fisher Scientific]). After a double-wash step with the same HBSS buffer without Fluo-4 AM, the plate was transferred to a FlexStation 3 (Molecular Devices, USA). Using the built-in automated pipetting system, kinetic measurement of fluorescence was performed (excitation = 485 nm, emission = 525 nm) before and after addition of I3A.

#### Cell viability studies

We used AlamarBlue Cell Viability Reagent (Thermo Fisher Scientific) to assess cell viability (metabolic activity) after treatment with I3A. The experiment was designed to mimic the conditions of the GLP-1 secretion studies. Cells were plated on a 96-well culture plate (30.000 cells/well) and cultured for 24 hours. Cell were then exposed to KRH-buffer containing 5.6 mM glucose and different concentrations of I3A during different times of exposure. In one experiment, cells were allowed to recover in culture medium. Before read-out, the buffer was replaced by 100 µL of 1:10 diluted AlamarBlue, and fluorescence was measured after 4 hours. The read-out of the negative control (cells kept in the standard culture medium during the entire experiment) was set as 100%, whereas 0% viability corresponded to the read-out from wells without seeded cells.

### Animals

Colon perfusions were performed in both male C57Bl/6JRj mice (Janvier labs, Le Genest-Saint-Isle, France; 25-30 grams) and male Wistar rats (Janvier labs; 300 grams). In the *in vivo* study we used female C57BI/6JRj mice (Janvier labs; 20 grams). Animals were handled in accordance with Danish legislation governing animal experimentation (1987). The studies were carried out with permission from the Danish Animal Experiments Inspectorate (2018-15-0201-01397) and the local ethics committee (P21-221 and P22-308). Animals were housed with a maximum of eight mice or four rats per cage in a standard 12-hour light, 12-hour dark cycle with free access to water and food.

### Perfusion studies

Non-fasted animals were anesthetized (mice: xylazine/ketamine; rats: hypnorm/midazolam) and placed on a heated operating table. After opening the abdominal cavity, the colon was separated from the small intestine, and flushed with saline to remove luminal content. Luminal perfusion was started and continued throughout the procedure (mice: 0.025 mL/min; rats: 0.15 mL/min). Sutures were placed around the small intestinal arterial arcades, celiac artery and renal arteries, and the spleen and stomach were removed. The upper abdominal aorta was ligated, and a catheter was inserted in the distal aorta. This procedure ensured selective perfusion of the colon. Perfusion buffer (PB) was a Krebs-Ringer bicarbonate buffer supplemented with 0.1% (w/v) bovine serum albumin (*cat. no. 1.12018.0500, Merck Ballerup, Denmark*), 5% (w/v) dextran T-70 (*Pharmacosmos, Holbaek, Denmark*), 3.5 mmol/L glucose, and 10 *μ*mol/L IBMX, pH 7.4). PB was heated to 37°C and gassed with a mixture of 95% O_2_ and 5% CO_2_ to maximally increase the oxygen concentration before entering the perfusion system (flow for mice: 1.5 mL/min; rats: 3 mL/min). Finally, a catheter was placed in the portal vein for the collection of venous effluent. Once the operation was complete, the colon was perfused for approximately 30 minutes before experiments commenced.

Each perfusion protocol started with a 10 min baseline period, followed by addition of I3A in different concentrations from either the luminal side (I3A diluted in saline) or the vascular side (I3A diluted in perfusion buffer). For luminal stimulation, the instillation rate was doubled for 5 minutes to ensure quicker filling of the colon with the stimulant. For vascular stimulation, an extra pump was added with the stimulant (infusion rate mice 0.075 mL/min; rats 0.15 mL/min) entering the perfusion line via a three-way stopcock. The venous effluent was collected at 1 min intervals, immediately chilled on ice and stored at -20°C until analysis.

We used radioimmunoassay using the C-terminal 89390-antibody to measure (amidated) GLP-1. This antibody is frequently used in perfusion models, as both rat and mice mainly secrete amidated GLP-1 (21).

### *In vivo* studies

A total of 32 female mice was divided into 4 groups (n=8): (1) Chow-diet + vehicle (CHOW); (2) Chow-diet + I3A (CHOW+I); (3) Fiber-free diet + vehicle (FF); and (4) Fiber-free diet + I3A (FF+I). Diet composition has been described previously (5). Each group was housed in a separate cage. During 10 days, twice daily, mice received 300 uL of I3A (2 mg/mL dissolved in MilliQ water) or vehicle (MilliQ water) via oral gavage. Each morning, before administration of treatment, animals and their food were weighed. Average food intake was calculated by dividing the total cage intake by the number of mice.

On the day of sacrifice (day 10), the mice were fasted for 4 hours from 7 AM. One hour prior to sacrifice, all animals received FITC-Dextran (*Sigma-Aldrich, cat. No. 60842-46-8*) through oral gavage (120μL/10g; 600mg/kg.bw) to measure (small) intestinal permeability. After the 4 hours fast, the mice were anesthetized (i.p. ketamine/xylazine). Total blood was taken from the inferior vena cava, after which the animal was euthanized by cutting up the diaphragm. Blood was transferred to pre-chilled EDTA-coated tubes, centrifuged (3000rpm, 4° C), and plasma was stored at -20°C until further processing. Finally, the small intestine and colon were removed and fixed in 4% paraformaldehyde before dehydration and paraffin embedding (22). Transverse sections of 4 µm were cut, stained with heamatoxylin and eosin and photographed using a light microscope connected to a camera. In sections from duodenum, jejunum, ileum and colon 20 well orientated crypts and villi were identified and measured using Zen Microscopy Software (Zeiss). Mucosal area quantification was conducted by subtracting the luminal circumference from the submucosal circumference.

Fasting plasma GLP-1 was measured using GLP-1 ELISA kit (*Mercodia, cat.no 10-1278-01*). To ensure that I3A did reach the colon, indole concentrations in fecal samples were measured using the Kovács reagent (*Sigma-Aldrich, cat. No. 129445*) based assay, as described here (23). FITC was measured by fluorescence analysis using an excitation wavelength of 485 nm and an emission wavelength of 528 nm in a SpectraMax iD3 multi-mode microplate reader (*Molecular Devices, San Jose, US*).

### Statistical analyses

Metabolomics were analyzed using unpaired t-test with correction for multiple testing using the method of Holm-Šídák. As the baseline GLP-1 levels varied between experimental days, *in vitro* data are expressed as relative to negative control for each experiment. Analyses were performed using linear mixed models, with drug exposure and biological replicates as fixed effect. *Post-hoc* testing of intervention compared to control was corrected using Holm-Šídák. For the *in vivo* study, results of all groups are shown. However, statistics are only given for the comparisons of CHOW versus FF, CHOW+I versus CHOW and FF+I versus FF, as these were the analyses of interest. Either one-way ANOVA or two-way ANOVA (for repeated measures over time) was used, with *post-hoc* Tukey. Statistics were performed using GraphPhad Prism 9. A two-sided *p*-value of <0.05 was considered statistically significant.

## Results

### Fiber-free diet lowered indole-3-carboxyaldehyde

**Figure 1:**
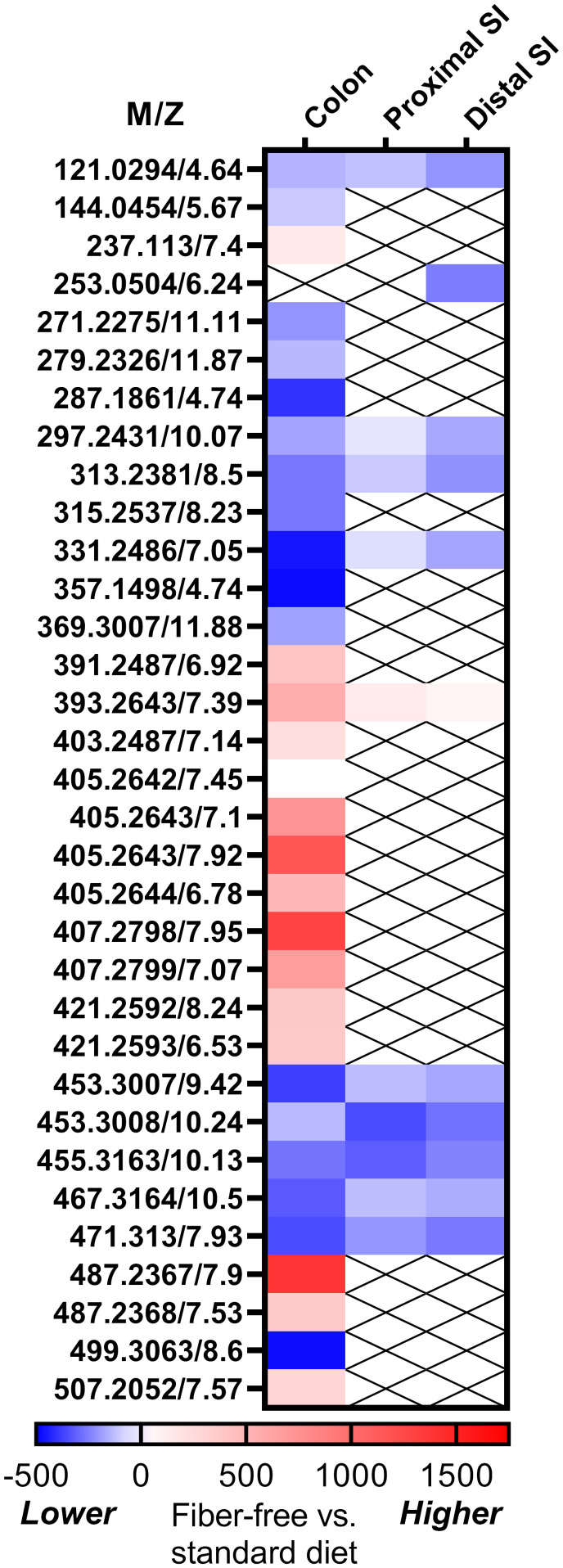
Results for fiber-free diet on intestinal metabolomics. Heat map showing the standardized mean difference between fiber-free diet and chow-diet in mice. Blue represents that the metabolite is lower in fiber-free diet, whereas red represents higher levels. The metabolites are given as m/z (mass to charge ratio), since we could not identify all of the metabolites.

We performed metabolomics on the intestinal content of mice fed a fiber-free or normal diet (**Figure 1**). In the distal small intestine, a metabolite likely to be dihydroxyflavone was significantly decreased in mice fed a fiber-free diet. In colonic contents, levels of several supposedly bile acids were higher in the mice fed a fiber-free diet. Levels of several fatty acids were lower, although the exact type could not be established. Importantly, in the colonic contents of fiber-free diet mice, I3A was significantly lowered. As I3A was the only metabolite which was decreased and identified with certainty, we decided to focus on the effects of supplementing this specific indole derivate.

### I3A influenced GLP-1 secretion *in vitro* in GLUTag cells

**Figure 2:**
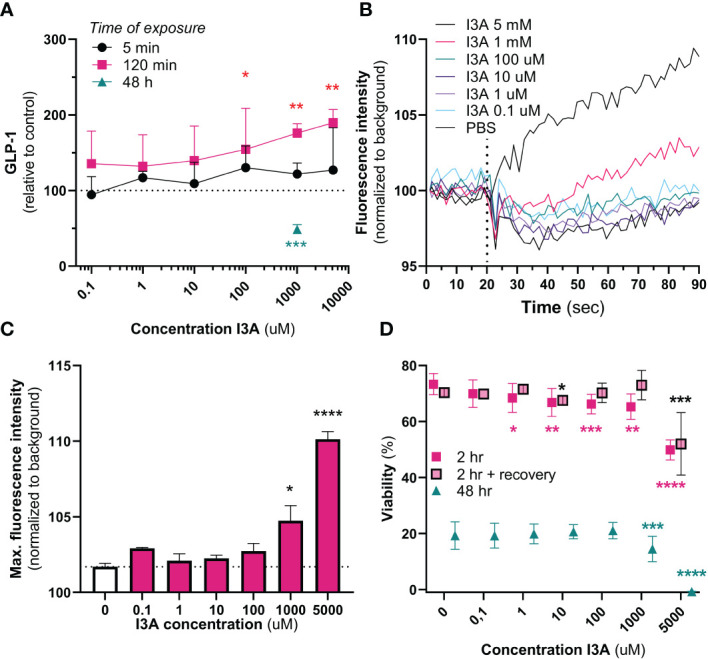
*In vitro* effects of I3A. Effects of I3A on different parameters in GLUTag cells. In all experiments, I3A was added to a buffer containing 5.6 mM glucose. **(A)**: GLP-1 secretion measured in response to different I3A-concentrations for an exposure time of 5 min (black circle, n=3), 120 min (red square, n=3) or 48 hours (green triangle, n=2). Levels were normalized to buffer containing no I3A. **(B)**: intracellular calcium mobilization, expressed as fluorescence intensity normalized to background over time, for different I3A concentrations. Interventions were given at 20 seconds (n=4). **(C)**: maximum peak of intracellular calcium mobilization, as seen in **(B)**, for each intervention. **(D)**: Viability measured in response to different I3A-concentratons after an exposure time of 2 hr (red square, n=4), 2 hours followed by 24 hours of recovery in culture medium (red square with black border, n=4) and 48 hours (green triangle, n=4). Levels were normalized to the viability in culture medium. Data are presented as mean ± standard error of the mean. Statistical analyses were performed using linear mixed models, comparing intervention to negative control. *p<0.05, **p<0.01, ***p<0.001 and ****p<0.0001.

Cumulative GLP-1 secretion was measured after 5 min, 240 min and 48 hours of exposure to different levels of I3A. Five minutes of exposure to I3A did not have any effect on GLP-1 secretion (overall *p*-value = 0.444) (**Figure 2A**). However, GLP-1 levels increased after 120 minutes of exposure to 1 mM (56.4% increase, *p*=0.0357) and 5 mM (73.5% increase, *p*=0.009), and tended to increase with 100 µM (52.1% increase, *p*=0.053). We additionally tested the effect of prolonged exposure, 48 hours, of GLUTag cells to I3A. Compared to control, I3A reduced GLP-1 secretion to 49.5% (*p*<0.001).

We next used another method to confirm the effect of I3A on GLUTag cells. Upon acute exposure, both 1 mM and 5 mM of I3A increased intracellular calcium mobilization, a final common pathway prior to GLP-1 secretion (**Figures 2B, C**). Compared to different doses of glucose, the effects of 1 and 5 mM were stronger (**Supplementary Figure 1**).

As supraphysiological doses of I3A might be toxic, we finally measured the effect of I3A on GLUTag cell viability (**Figure 2D**). Except for 0.1 µM, all tested doses appeared to temporarily reduce viability upon 2 hours of exposure. However, only the 5 mM seriously reduced viability after recovery of the cells in culture medium. After 48 hours of exposure, only 1 mM and 5 mM significantly reduced viability.

### I3A had no effect on the isolated perfused colon

**Figure 3:**
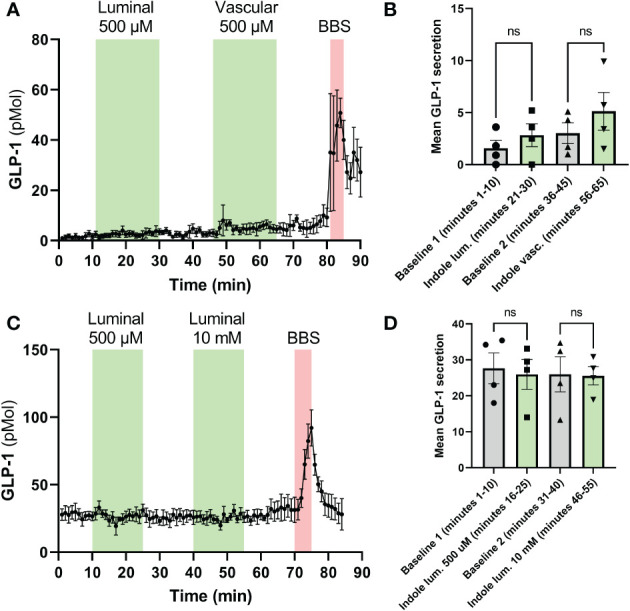
Effects of I3A on perfusion models. The effects of I3A on GLP-1 secretion were assessed in isolated rat colon perfusion models. Both luminal and vascular application of I3A were studied. In the left **(A, C)**, the hormone levels are shown minute-by-minute over the duration of the experiment. The green areas represent the time during which I3A was perfused, the red areas represent the positive control (BBS, bombesin). In the right **(B, D)**, averages of the last 10 minutes of each baseline and intervention are shown. **(A)**: GLP-1 levels in rat colon perfusions; **(B)**: 10-minute average for experiments in **(A)**. **(C)**: GLP-1 levels in mouse colon perfusions; **(D)**: 10-minute average for experiments in **(C)**. All data are given as mean ± standard error of the mean. Statistics were performed on the averages using paired t-tests. NS, not significant.

In mice, neither luminal nor vascular infusion of I3A stimulated GLP-1 at 500 µM. We then assessed whether there might be species-specific differences by perfusing rat colons. Using 500 µM as in mice, but also a 10 mM concentration, infusion of I3A had no effect on GLP-1 secretion (**Figure 3**). Importantly, experiments were successful as demonstrated by the increase in hormone levels after bombesin infusion, and normal pressure during the perfusion (data not shown).

### I3A did not counteract the effects of fiber-free diet *in vivo*

**Figure 4:**
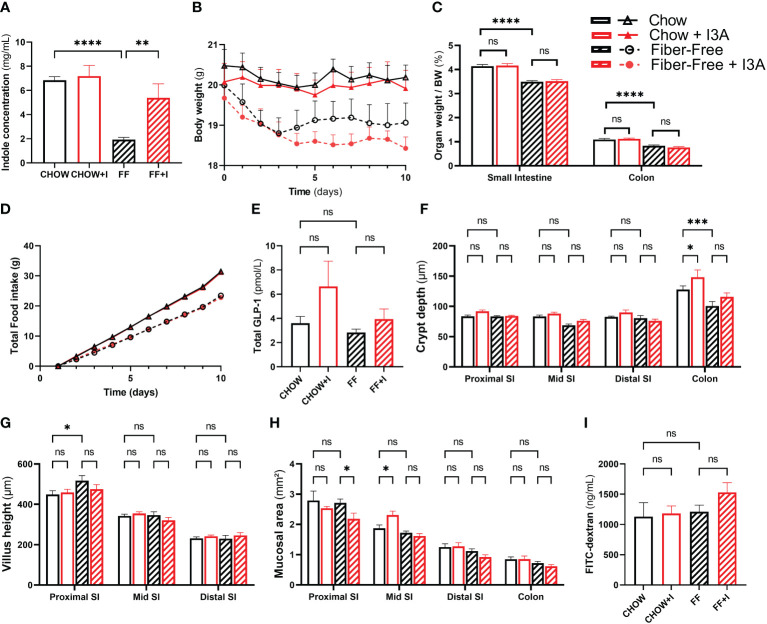
Effects of *in vivo* administration of I3A during fiber-free diet feeding. Female C57Bl/6JRj mice were fed chow or fiber-free (FF) diet, and received either I3A (+I) or control, during 10 days (n=8 per group). Mice were group-housed per intervention. **(A)**: fecal indole concentrations as measured using Kovac’s assay; **(B)**: body weight of individual mice during the study period; **(C)**: organ weight after euthanasia, expressed as relative to total body weight; **(D)** daily total food intake was recorded per cage. As all animals per intervention were housed in one cage, these data do not have error bars; **(E)**: fasting total GLP-1 levels in plasma, measured using the Mercodia total GLP-1 assay; **(F)**: crypth dept was measured using light microscopy on H&E colored sections of the proximal small intestine, mid small intestine, distal small intestine and colon; **(G)**: villus height; **(H)**: total mucosal area; and **(I)**: intestinal permeability as assessed using FITC-Dextran. Bars represent means ± standard error of the mean. Statistics were done using one-way or two-way ANOVA were appropriate, only comparing CHOW to FF, CHOW+I to CHOW and FF+I to FF. Statistical significance is indicated as *p<0.05, **p<0.01, ***p<0.001 and ****p<0.0001. NS, not significant.

First, to ensure that the intervention was successful, we measured total indole concentration in fecal samples. The mice in the FF group had lower indole concentrations (4.9 mg/mL [SEM 1.06], *p*=0.0001), confirming the earlier observations. Compared to FF, FF+I had 3.46 mg/mL (SEM 1.06) higher indole levels (*p*=0.0098) (**Figure 4A**), confirming that I3A was present.

After 10 days, mice fed FF had a numeric reduction in body weight, compared to those fed CHOW. In the FF group, body weight was 1.1 g (SEM 0.55) lower (p = 0.21) compared to CHOW (**Figure 4B**). Addition of indoles to the fiber-free diet did not change this, but led to a further decrease in weight (1.8 g [SEM 0.55], p=0.018). FF diet resulted in significant reduction in the weight of the small intestine and colon compared to CHOW (**Figure 4C**). There was no difference between FF and FF+I.

Food intake was different among the treatment groups (**Figure 4D**). Mice in FF ate 8 g less than CHOW mice, as seen before (5). Those in FF+I ate 0.6 g less than FF, and 8.6 g less then CHOW. Plasma levels of GLP-1 did not differ among any of the groups (**Figure 4E**). However, when combining data from the non-indole groups (CHOW and FF) and the indole-treated groups (CHOW+I and FF+I), there was a trend towards an increase in GLP-1 secretion in the indole-treated group (increase of 1.8 pmol/L [SEM 1.0], *p*=0.079, *data not shown*). None of the dietary interventions induced statistically significant changes in crypt depth, mucosal area or villus height (**Figures 4F–H**). Finally, intestinal permeability was similar among all groups (**Figure 4I**).

## Discussion

In the current study, we show for the first time how a fiber-free diet alters the intestinal metabolome of C57Bl/6JRj mice. Importantly, we report the intestinal bacteria-derived tryptophan metabolite I3A to be decreased in the colonic content. I3A stimulates GLP-1 secretion *in vitro*, but did not show any beneficial effect on intestinal health when administered *in vivo* and did not restore the negative effects of fiber-free diet on body weight or intestinal morphology.

The current study was inspired by our previous finding that a fiber-free diet disrupts intestinal weight, L-cell secretion, and intestinal integrity (5). Here, we performed untargeted metabolomics on intestinal contents collected in the aforementioned study, to explore changes in the metabolome caused by fiber-free diet. While several metabolites could be identified with certainty, the only identified metabolite that was significantly reduced – and could thus be supplemented in further experiments - was I3A. Although we are the first to demonstrate that this metabolite decreases in fiber-free diet, others have shown that increases in dietary fibers cause increases in I3A levels (24).

I3A is considered an indole-containing compound, and is produced in the intestine by bacterial synthesis from the essential amino acid L-tryptophan. Indole and indole-containing compounds all share a common backbone (a six-membered benzene ring fused to a five-membered pyrrole ring), yet they differ in side-chains. The faith of L-tryptophan depends on the presence of individual bacterial enzymes. For example, tryptophanase-expressing bacteria generate indole, whereas tryptophan aminotransferase leads to indole-3 pyruvate. While the involved enzymes remain unknown, indole-3-carboxyaldehyde is produced by *Lactobacilli*. As such, each unique microbiota will produce different concentrations of indole-derivates and studies have shown that a fiber-free diet reduces *Lactobacillus* presence in the intestinal microbiota (25), consistent with our finding of reduced I3A levels in colonic content.

We hypothesized that the reduction in I3A could explain some of the adverse effects of fiber-free diet, and that these effects might be mediated by reduced secretion of GLP-1 and GLP-2 (which are co-secreted (26)). This idea was based on previous studies where indole induced secretion of GLP-1 in GLUTag cells (19), and moreover improved glucose tolerance and increased ileal *Cgc*-expression in mice (probably accompanying increased GLP-1 secretion) (27). Moreover, GLP-1 and especially GLP-2 are involved in mucosal health and inflammation (28). However, up to now, the effects of I3A on GLP-1 secretion were unknown.

In GLUTag cells, calcium mobilization, which is one of the crucial intracellular steps toward GLP-1 secretion, increases acutely upon exposure to I3A. Five minute exposure to I3A had no statistical effect on GLP-1 secretion (although a tendency was observed), while prolonged exposure for 2 hours was associated with an increase in GLP-1. Secretion dropped to half of that of the negative control when cells were exposed to I3A for multiple days. A similar pattern on GLP-1 secretion was seen with indole in GLUTag cells (19), where higher concentrations of indole stimulated GLP-1 secretion initially, but inhibited secretion after prolonged exposure. Mechanistically, short time exposure reduced the passage of current through voltage-gated potassium channels, leading to prolonged activation of Ca^2+^-channels, and enhanced GLP-1 release. Prolonged exposure, however, inhibited NADH dehydrogenase, which resulted in lower intracellular ATP availability, which hyperpolarizes cells and reduces GLP-1 release (19). Although we did not test this here, these mechanisms could also apply for I3A. Alternatively, I3A could be toxic to GLUTag cells at higher doses for prolonged periods of time. Unfortunately, the AlamarBlue assay we employed detects metabolically active cells, and will give decreased signals with less viable cells, but also when cells are less metabolically active (such as decreased NADH dehydrogenase activity). Moreover, the KRH buffer which we used for these experiments is not the same as culture medium, and causes a starvation state that decreases metabolic activity.

In colon perfusion models, no effect on GLP-1 secretion was seen during I3A administration. We tested both luminal and vascular administration and included mice and rats to exclude species-dependent effects. Similarly, when given as daily gavage to mice fed a fiber-free diet, I3A did not affect plasma GLP-1 levels. The discrepancy between the *in vitro* study and the studies in animals could be dose-related, as the direct exposure to L-cells *in vitro* is not comparable to the dilution which occurs in the animal experiments. We were, however, bound by the limited solubility of I3A in water. While I3A was more easily dissolved in DMSO and ethanol, these solvents are toxic to the used models, and we could therefore, not try higher doses. Another hypothesis could be that the microbiota present in the animals degrades the I3A before it could stimulate L-cells. Finally, as there was a trend towards an increase in GLP-1 levels with indole-treatment when the chow- and fiber-free fed animals were combined, there could be a lack of power in the *in vivo* study.

Regardless of the effects on GLP-1, I3A might reverse the effects of fiber-free diet. To assess this, we fed mice either chow or a fiber-free diet, and treated them with either I3A or saline by oral gavage. Importantly, the fiber-free diet yielded similar results as in our previous study (5), with lower body weight and food intake. However, I3A was not able to reduce any of the effects of fiber-free diet.

This lack of effect contrasts with previous studies, where I3A given to C57Bl/6J-mice reduced intestinal permeability (29) and reduced inflammation (30). Given this discrepancy we were concerned that the orally administered I3A did not reach the colon in our study. However, using Kovac’s assay, we observed an increase in fecal indole levels. One caveat is that this assay measures indoles as a group, and any conversion of I3A to other indoles cannot be distinguished. Another hypothesis for the lack of effect could be that the exposure time was not long enough. Moreover, we gave the I3A in the morning time, which is when mice normally start fasting, while physiologically, intestinal microbiota generate I3A when tryptophan arrives with the diet. When comparing our neutral findings to the previous positive studies with I3A, a crucial difference are the used models. Previously, dextran sulfate sodium (DSS) was used to induce intestinal inflammation, leading to a very different clinical condition compared to that seen with fiber-free diet. Also, I3A was given differently (spray-dried, or in higher doses) (29, 30). Finally, one could argue that I3A simply is not effective in improving the negative effects of fiber-free diet.

In conclusion, similar to indoles, I3A influences GLP-1 secretion *in vitro*, yet has no evident beneficial effects on intestinal health during fiber-free feeding when administered *in vivo*.

## Data availability statement

The raw data supporting the conclusions of this article will be made available by the authors, without undue reservation.

## Ethics statement

The animal study was approved by Danish Animal Experiments Inspectorate and the local ethics committee. The study was conducted in accordance with the local legislation and institutional requirements.

## Author contributions

MS: Formal analysis, Investigation, Methodology, Writing – original draft, Writing – review & editing. SD: Investigation, Methodology, Writing – original draft, Writing – review & editing. JEH: Conceptualization, Investigation, Methodology, Writing – review & editing. AD: Data curation, Writing – review & editing. IM: Conceptualization, Investigation, Supervision, Writing – review & editing. JJH: Funding acquisition, Methodology, Supervision, Writing – review & editing. HK: Conceptualization, Funding acquisition, Investigation, Methodology, Project administration, Resources, Writing – original draft, Writing – review & editing.
