# Supplementary material for: Indole-3-carboxyaldehyde does not reverse the intestinal effects of fiber-free diet in mice
Source: Front Endocrinol (Lausanne). 2024 Mar 22;15:1362711. doi: 10.3389/fendo.2024.1362711 (PMC10995233; doi:10.3389/fendo.2024.1362711)
Supplement: Supplementary file 1 [file Image_1.pdf]

Supplementary Figure 1: In vitro calcium mobilization of I3A versus glucose.

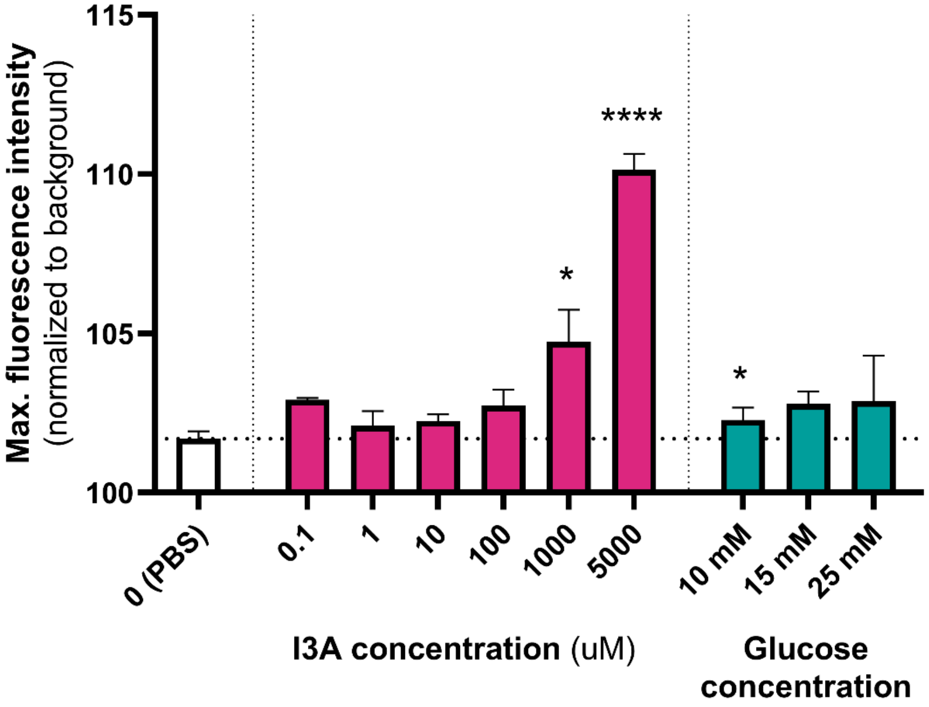

Legend: Maximum peak of calcium mobilization (fluorescence intensity normalized to background). The PBS (clear bar) and I3A (red bars) are identical data as in Figure 2B, but here included is also glucose (green bars).

Data are presented as mean ± standard error of the mean. Statistical analyses were performed using linear mixed models, comparing intervention to negative control. *p<0.05, **p<0.01, ***p<0.001 and ****p<0.0001.
